# Galectin-3 and cyclin D1 expression in non-small cell lung cancer

**DOI:** 10.1186/1756-9966-30-101

**Published:** 2011-10-24

**Authors:** Monika Kosacka, Paweł Piesiak, Aneta Kowal, Marcin Gołecki, Renata Jankowska

**Affiliations:** 1 Chair and Department of Pulmonology and Lung Cancer, Silesian Piasts University of Medicine in Wroclaw, Poland, 53-439 Wroclaw, ul. Grabiszynska 105

**Keywords:** galectin-3, cyclin D1, non-small cell lung cancer, prognostic factor

## Abstract

**Introduction:**

Lung cancer is a major cause of mortality and morbidity worldwide. Galectin-3 is multifunctional protein, which is involved in regulation of cell growth, cell adhesion, cell proliferation, angiogenesis and apoptosis. Cyclin D1 together with other cyclin plays an important role in cell cycle control. Cyclin D1 regulates the G1-to-S phase transition. The aim of this study was the evaluation of correlations between clinicopathological findings and cyclin D1 and galectin-3 expression in non-small cell lung cancer (NSCLC). We wanted also to analyze the prognostic value of cyclin D1 and galectin-3 expression. Moreover we tried to evaluate the correlations between galectin-3 and cyclin D1 expression in tumor tissue.

**Materials and methods:**

We used the immunochemistry method to investigate the expression of galectin-3 and cyclin D1 in the paraffin-embedded tumor tissue of 47 patients (32 men and 15 women; mean age 59.34 ± 8.90). years. We used monoclonal antibodies to cyclin D1 (NCL-L-cyclin D1-GM clone P2D11F11 NOVO CASTRA) and to galectin-3 (mouse monoclonal antibody NCL-GAL3 NOVO CASTRA).

**Results:**

Galectin-3 expression was positive in 18 cases (38.29%) and cyclin D1 in 39 (82.97%). We showed only weak trend, that galectin-3 expression was lower in patients without lymph node involvement (p = 0.07) and cyclin D1 expression was higher in this group (p = 0.080). We didn't reveal differences in cyclin D1 and galectin-3 expression in SCC and adenocarcinoma patients. We didn't demonstrated also differences in galectin-3 and cyclin D1 expression depending on disease stage. Moreover we analyzed the prognostic value of cyclin D1 expression and galectin-3 in all examinated patients and separately in SCC and in adenocarcinoma and in all stages, but we didn't find any statistical differences. We demonstrated that in galectin-3 positive tumors cyclin D1 expression was higher (96.55% vs 61.11%, Chi^2 ^Yatesa 7.53, p = 0.0061) and we revealed negative correlation between cyclin D1 and galectin-3 expression (R Spearman -0.458, p = 0.0011). In squamous cell lung cancer we didn't observed correlations between these both examinated markers (R = -0.158, p = 0.460), and in adenocarcinoma the negative correlation was very strong (R = -0.829 p = 0.000132).

**Conclusions:**

We didn't reveal any important correlations between clinicopathological findings and galectin-3 and cyclin D1 expression and in non small cell lung cancer. We didn't observed also prognostic value of cyclin D1 or galectin-3 expression. But we showed higher cyclin D1 expression in galectin-3 negative tumor tissues. We revealed also differences in correlations between galectin-3 and cyclin D1 expression in two main histopathological types of NSCLC.

## Introduction

Lung cancer is the most commonly diagnosed cancer as well as the death cause in males. Among females it is the fourth cancer worldwide and the second leading cause of cancer death. Although in developed countries consists the second common neoplasm in females [1,2]. The overall 5-year survival rates of lung cancer patients remain relatively poor. EUROCARE-4 the large population study on survival of adult Europeans with cancer, reported that mean age-adjusted 5-year survival for lung cancer was 12.5%. This survival rate seems to be very low especially in comparison with survival in another carcinomas (colorectal-53.8%, breast-78.9%, prostate-75.7%, ovarian-36.3%) [3]. Currently the most powerful prognostic tool in lung cancer is the stage of disease. Differing survival outcomes among patients within a stage suggests the existence of other tumor factors affecting prognosis. Such factors could potentially be used to further classify patients into groups according to sub-stages that may be treated differently.

Galectin-3 belongs to the evolutionary conserved family of 15 carbohydrate-binding proteins that are widely distributed in normal and neoplasmatic cells [4]. Galectin-3 is a 31 kDa molecule, that consists of three domains: a NH2 terminal domain, a repetitive collagen-like sequence rich in glycine, proline and a COOH-terminal carbohydrate recognition domain (CRD, lectin domain)[5]. CRD is responsible for the specificity of galectins for saccharides [6]. This intracellular and extracellular lectin is able to interact with many molecules including glycoproteins, cell surface molecules and extracellular matrix proteins [5]. Galectin-3 is multifunctional protein, which is involved in regulation of cell growth, cell adhesion, cell proliferation, angiogenesis and apoptosis. Intracellular galectin-3 could inhibit cell apoptosis induced by chemotherapy agents such as cisplatin and etoposide [7]. The connection with cancer progression and oncological drug resistance indicate that galectin-3 seems to be promising target for the development of novel oncological therapeutic strategies [6,7]. Uncontrolled cell proliferation is the hallmark of malignant tumors that is why the evaluation of the prognostic significance of the expression of proteins involved in regulation of cell proliferation remains promising. Cellular proliferation is regulated by protein complexes composed of cyclins and cyclin-dependent kinases (cdks). Five major families of cyclins (termed A, B, C, D, and E) have been isolated and characterized. Cyclin D1 reaches it peak of synthesis and activity during the G1 phase, and is believed to regulate the G1-to-S phase transition [8,9]. Cyclin D1 plays a role in DNA repair. Cyclin D1 could bind directly RAD51, a recombinase that drives the homologous recombination process [10]. Cyclin D1 gene is located in the chromosome 11q13 [11]. The expression of cyclin D1 and other cyclins has been often evaluated in many cancers and its prognostic value is disputable. In esophageal squamous cell carcinoma and hepatocellular carcinoma the expression of CyclinD1 has been reported to be associated with poor outcomes [12-14].

The aim of this study was the evaluation of correlations between clinicopathological findings and cyclin D1 and galectin-3 expression in non-small cell lung cancer. We wanted also to analyze the prognostic value of cyclin D1 and galectin-3 expression. Moreover we tried to evaluate the correlations between galectin-3 and cyclin D1 expression in tumor tissue.

## Materials and methods

The 47 patients with non-small cell lung cancer (32 men and 15 women) were evaluated. The mean age of the patients was 59.34 ± 8.90 years. All patients had undergone the surgical treatment (lobectomy, bilobectomy, pneumonectomy or diagnostic thoracotomy). The histopathologic diagnosis was squamous cell carcinoma in 24 patients, adenocarcinoma in 15 patients, large cell carcinoma in 4 patients and non- small cell lung cancer of unspecified type in 4 patients. Based on the TNM staging system: 17 patients were in stage I (including IA-5 persons, IB-12), 8 in II (IIA- 1, IIB-7), 16 in III (IIIA-13, IIIB-3) and in 6 IV.

Twenty-one patients received chemotherapy-treatment, in this group 12 persons neoadjuwant chemotherapy.

In all patients the 24 month survival has been evaluated. Twenty seven (57.45%) patients were alive and 20 (42.55%) died. The average survival time was 18.91 ± 7.14 months.

The work has been approved by the appropriate ethical committees related to the institution.

### Immunohistochemistry

Formalin -fixed well preserved tumor tissue blocks from surgically resected lung cancer specimens were used for immunohistochemical study. The 4 μm-sections of formalin -fixed tissues were mounted on silanized slides, deparaffinized in xylene and rehydrated through serial baths of alcohol to water. The hydrated sections were treated in 3% hydrogen peroxide for 10 minutes to eliminate endogenous peroxidase activity and washed in phosphate-buffered saline (PBS).

The primary antibodies used in this study were:

Galectin-3 mouse monoclonal antibody NCL-GAL3 NOVO CASTRA and Cyclin D1 monoclonal antibody (NCL-L-CYCLIN D1-GM clone P2D11F11 NOVO CASTRA).

The monoclonal antibody-treated slides were raised in PBS solution and incubated with a biotinylated secondary antibody (LSAB^R^+ Kit DAKO). The slides were washed in PBS and then incubated with an avidin-biotin-peroxidase complex (LSAB^R^+ Kit, DAKO K 0675) for 15 minutes. After washing with PBS, a chromogenic reaction was developed by incubating with 3,3-diaminobenzidine tetrahydrochloride (DAB+, Liquid K 3486 DAKO).

Positive staining appeared as brown cell plasma or nucleus. The galectin-3 and cyclin D1 expression was described as positive if more than 10% of cells were stained.

### Statistical method

Statistical analysis was performed using the CSS Statistica for Windows (version 5.0). Chi-square test was used among two or multiple groups. Differences between samples were considered significant at p < 0.05. Survival curves were constructed using Kaplan-Meier method.

## Results

**Figure 1:**
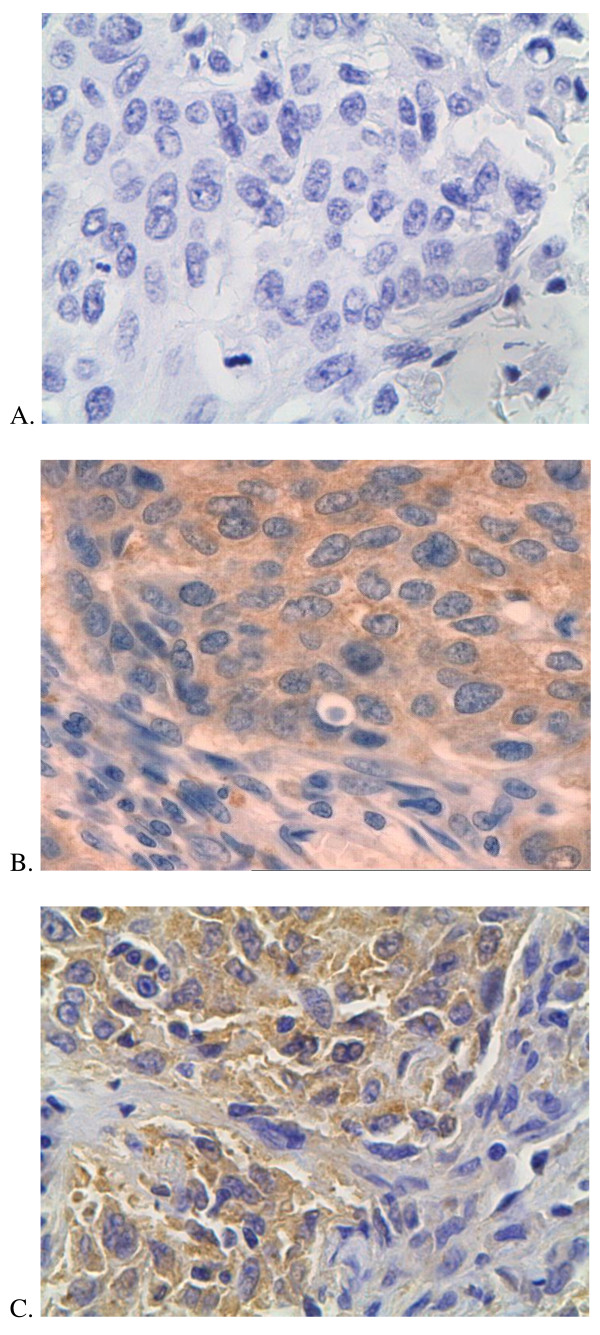
**Immunohistochemical staining**. A. negative immunostaining; B.positive cytoplasmatic cyclin D1 immunostaining; C.positive cytoplasmatic galectin-3 immunostaining.

The galectin-3 expression was revealed in 18 cases (38.29%). Only cytoplasmatic staining war observed. Figure 1 shows pictures of immunohistochemical staining (Figure 1).

In squamous cell carcinoma (SCC) galectin-3 expression was positive in 11 from 24 tumor specimens (45.83%), in adenocarcinoma in 4 from 15 (26,67%), in large cell carcinoma in 2 from 4 (50%) and in non- small cell lung cancer of unspecified type in 1 from 4 (25%). We compared galectin-3 expression in two main histopathogical types: SCC and adenocarcinoma, but any statistical significant differences were revealed (Chi^2 ^Yatesa 0.74, p = 0.390). We didn't perform comparison in another histopathological types because of the small numerous of the groups.

In stage I galectin-3 was positive in 3 from 17 tumor specimen (17.65%), in stage II in 5 from 8 (62.5%), in stage III 7 from 16 (43.75%) and in stage IV in 3 from 6 (50%). We didn't reveal differences in galectin-3 expression depending on disease stage. We wanted also to analyze if chemotherapy before surgical treatment (neoadjuwant therapy) could change galectin-3 expression in tumour tissue, that is why we performed comparison of galectin-3 expression in patients, who received neoadjuwant chemotherapy and patients, who didn't receive chemotherapy before surgery. In the first group galectin-3 expression was positive in 5 tumour tissues from 12 (41.6%) and in the second group in 13 from 35 (37.14%). The difference was not significant. Moreover we compared galectin-3 expression in patients with lymph nodes metastases (N1 and N2) and in patients without (N0). In patients with lymph node metastases galectin-3 expression was revealed in 13 from 25 cases (52%), and without lymph node metastasis in 5 from 22 (22.7%). In Chi^2 ^test the difference was significant (p = 0.039), but in Chi^2 ^Yatesa test there was only tendency (p = 0.07).

**Figure 2:**
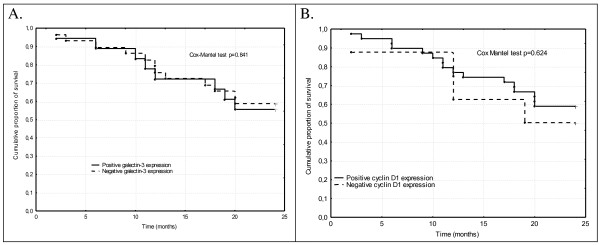
**Cumulative proportion of survival Kaplan- Meier in all patients with non-small cell lung cancer according to: A galectin-3 expression; B. cyclin D1 expression**.

We analyzed the prognostic value of galectin-3 expression in all patients with NSCLC and separately in patients with SCC and adenocarcinoma, and separately in every stage, but we didn't find any statistical significant differences (Table 1 and Figure 2).

**Table 1:** The comparison of 24 months survival and galectin-3 expression in selected groups of patients.

Survival	Positive galectin-3 expression n (%)	Negative galectin-3 expression n (%)	Chi^2^ Yatesa	p	Cox Mantel
**All examinated patients with NSCLC**					

< 24 months	8 (44.44%)	12 (41.38%)	0.01	0.922	0.841
	
≥ 24 months	10 (55.56%)	17 (58.62%)			

**The patients with squamous cell carcinoma**					

< 24 months	5 (45.45%)	5 (38.46%)	0.00	0.944	0.612
	
≥ 24 months	6 (54.55%)	8 (61.54%)			

**The patients with adenocarcinoma**					

< 24 months	2 (50%)	6 (54.55%)	0.18	0.667	0.695
	
≥ 24 months	2 (50%)	5 (45.45%)			

**Stage I**					

< 24 months	1 (33.33%)	2 (14.29%)	0.00	0.960	0.434
	
≥ 24 months	2 (66.66%)	12 (85.71%)			

**Stage II**					

< 24 months	2 (40%)	3 (100%)	0.89	0.345	0252
	
≥ 24 months	3 (60%)	0 (0%)			

**Stage III**					

< 24 months	2 (28.57%)	5 (55.56%)	0.33	0.567	0.275
	
≥ 24 months	5 (71.43%)	4 (44.44%)			

**Stage IV**					

< 24 months	3 (100%)	2 (66.67%)	0.00	1.00	0.341
	
≥ 24 months	0 (0%)	1 (33.33%)			

Thirty-nine of 47 (82.97%) tumor tissue specimens were positive for cyclin D1. Only cytoplasmatic staining were observed (Figure 1). We analyzed cyclin D1 expression in two main histopathological types. In SCC positive cyclin D1 expression was detected in 21 from 24 cases (87.5%) and in adenocarcinoma in 12 from 15 (80%). There was no significant differences in cyclin D1 expression (Chi^2 ^Yatesa 0.03; p = 0.860). We didn't reveal also differences in cyclin D1 expression in male and female (p = 0.964). In stage I cyclin D1 was positive in all 17 tumor specimen (100%), in stage II in 4 from 8 (50%), in stage III 14 from 16 (87.5%) and in stage IV in 4 from 6 (66.7%). We didn't reveal differences in cyclin D1 expression depending on disease stage. The cyclin D1 was compared also in patients with lymph node metastasis (N1 or N2) and in patients without lymph node involvement (N0). In patients with N0 cyclin D1 was positive in 21 from 22 cases and in patients with N1 or N2 cyclin was positive in 18 from 25. In Chi^2 ^test the difference was significant (Chi^2 ^4.46; p = 0.032), but in Chi^2 ^Yatesa test there was only tendency (3.05, p = 0.080)

We analyzed the prognostic value of cyclin D1 expression in all patients with NSCLC and separately in patients with SCC and adenocarcinoma, and separately in every stage, but we didn't find any statistical significant differences (Table 2 and Figure 2).

**Table 2:** The comparison of 24 months survival and cyclin D1 expression in selected groups of patients.

Survival	Positive Cyclin D1 expression n (%)	Negative Cyclin D1 expression n (%)	Chi^2^ Yatesa	p	Cox Mantel
**All examinated patients with NSCLC**					

< 24 months	16 (41.03%)	4 (50%)	0.01	0.940	0.624
	
≥ 24 months	23 (58.97%)	4 (50%)			

**The patients with squamous cell carcinoma**					

< 24 months	8 (38.10%)	2 (66.67%)	0.10	0.754	0.234
	
≥ 24 months	13 (61.90%)	1 (33.33%)			

**The patients with adenocarcinoma**					

< 24 months	7 (58.33%)	1 (33.33%)	0.02	0.897	0.396
	
≥ 24 months	5 (41.67%)	2 (66.67%)			

**Stage II**					

< 24 months	4 (100%)	1 (25%)	2.13	0.144	0.076
	
≥ 24 months	0 (0%)	3 (75%)			

**Stage III**					

< 24 months	6 (42.86%)	1 (50%)	0.33	0.567	0.544
	
≥ 24 months	8 (57.14%)	1 (50%)			

**Stage IV**					

< 24 months	3 (75%)	2 (100%)	0.15	0.698	0.085
	
≥ 24 months	1 (25%)	0 (0%)			

We decided also to compare correlations between cyclin D1 and galectin-3 expression. In galectin-3 positive tumors cyclin D1 was positive in 11 from 18 (61.11%) and in galectin-3 negative was positive in 28 from 29 (96.55%). The difference was statistical significant (Chi^2 ^Yatesa 7.53, p = 0.0061) and the Spearman's correlation coefficient confirmed negative correlation between cyclin D1 and galectin-3 expression (R Spearman -0.458, p = 0.0011). We tried also to compare correlations between examinated markers in both main histopathological types. In squamous cell lung cancer we didn't observed correlations between these both examinated markers (R = -0.158, p = 0.460), and in adenocarcinoma the negative correlation was very strong (R = -0.829 p = 0.000132).

## Discussion

Many studies indicate on enorm potential of immunohistochemical method in better understanding of the carcinogenesis and in searching of prognostic factors in lung cancer [15-17].

The importance of galectin-3 expression remains disputable. It seems to be interesting that galectin-3 expression could play different roles in another carcinomas. The expression of galectin-3 is associated with tumor invasion and metastatic potential in head, neck, thyroid, gastric and colon cancers. In contrast, for some tumours such as breast, ovarian and prostate cancer the expression of galectin-3 is inversely correlated with metastatic potential [5].

Szoeke and co-workers investigated the prognostic value of growth/adhesion-regulatory lectins in stage II non-small cell lung cancers. In examinated group of 94 patients they showed poorer prognosis for the galectin-1 and galectin-3-expressing tumor in the univariate survival examination and in the multivariate analysis for the galectin-3 positive tumours. Moreover they suggest that in tumours expressing and binding galectin-3, the distance between the tumour cells is of prognostic significance and an increase in the microvessel volume fraction points to a poorer survival rate [18].

Our study doesn't confirm the prognostic value of galectin-3 expression. This could be connected with relative small and heterogenous group of patients. Moreover the reason could be related also to the staining patterns. We revealed only cytoplasmatic staining and this is the main pattern of galectin-3 expression. Nuclear and cytoplasmatic co-expression are observed relative rare [19], but two variants of galectin-3 are known: a phosphorylated and a non-phosphorylated form. Phosphorylation is a requirement for its nuclear export [20]. Hubert et co-workers studied the intracellular distribution of galectin-3 in mouse 3T3 fibroblasts and observed that proliferating cells showed higher expression of galectin-3 in the nucleus than in cytoplasm, but quiescent cells predominantly expressed galectin-3 in cytoplasm [21].

We observed, that galectin-3 expression was higher in patients with lymph node metastases (tendency in Chi^2 ^Yatesa test and statistical significance in Chi^2 ^test). Others studies confirm that increased expression of galectins family members, could correlate with elevated invasiveness. It has been showed in experimental study, that increased galectin-1 expression was associated with high levels of invasion in lung adenocarcinoma and oral squamous cell carcinoma lines [22]. Wu et al. demonstrated in 37 colon cancer patients, that galectin-3 expression was significantly higher in tumors with lymph node metastasis [23]. Liang and co-workers showed in non small cell lung cancer, that not only galectin-3 expression in tumor tissue could be connected with occurrence of metastasis, but also higher serum level of galectin-3 could indicate on increased risk of occult metastasis [24].

The correlation between cyclin D1 expression and clinicopathological findings as well as prognosis remains disputable. Mishina and al. showed that the 5-year survival was better in patients with cyclin D1 positive tumours (89% vs 64%), and cyclin D1 expression tended to be a favourable prognostic factor in univariate analysis (p = 0.08) [25].

Ayeda and al. observed in 98 patients with resected stage I and II NSCLC, that patients with cyclin D1-positive tumors had shorter survival than those with cyclin D1-negative tumors (5-year survival rates, 48% vs 74%; p = 0.006) [26]. Other authors didn't confirm the prognostic value of cyclin D1 expression in resectable non small cell lung cancer [27].

We revealed only weak tendency that cyclin D1 expression was higher in patients without lymph node involvement. The correlations between cyclin D1 expression and clinicopathological findings remain disputable. Some authors indicate, that cyclin D1 had significantly higher positive results in patients with poorly differentiated carcinoma, in presence of vascular invasion and visceral pleural invasion [26].

We revealed higher cyclin D1 expression in galectin-3 negative tumors (96.55% vs 61.11%, p = 0,0061) and negative correlation between cyclin D1 and galectin-3 expression (R Spearman -0.458, p = 0.0011). These results were surprising for us, because some studies indicate on positive correlations between these both examinated markers in selected carcinoma types. Ferrazzo and al. demonstrated in adenoid cystic carcinoma of salivary glands, that cyclin D1 expression was correlated with cytoplasmatic and nuclear galectin-3 expression, what could suggests that galectin-3 may play a role in cellular activation through cyclin D1 activation, but these authors observed in adenoid cystic carcinomas predominately nuclear galectin-3 expression [28]. Acikalin et al. showed correlation between galectin-3 and cyclin D1 expression in undifferentiated nasopharyngeal carcinoma [29].

However the number of studies, which evaluated correlations between galectin-3 and cyclin D1 expression is limited and we didn't find any studies performed in lung cancer tissue. Experimental studies in human breast epithelial cells indicate that galectin-3 could down-regulate the cyclin E and cyclin A expression [30]. The same authors suggested that galectin-3 up-regulated cyclin D1 expression, but they observed also that galectin-3 up-regulation of cyclin D1 expression enhanced in suspension cultures. From the other hand it is known that cell adhesion is required for the induction and translation of cyclin D1 mRNA, moreover in cyclin D1 expression play role different factors [31]. That is why experimental results on cultures could differ from clinical studies on tumor tissue. Moreover as mentioned before galectin-3 expression could play different roles in different carcinomas types [5].

We revealed also differences in correlations between galectin-3 and cyclin D1 expression in two main histopathological types of NSCLC. In squamous cell lung cancer we didn't observed correlations between these both examinated markers, and in adenocarcinoma the negative correlation was very strong. We didn't find any similar works comparing correlations between galectin-3 and cyclin D1 expression, but the results were not so surprising for us. The differences between these both histopathological types are well known, beginning from changes in incidence, through the differences in molecular biology and ending in various therapeutic strategies [32].

## Conclusions

We didn't reveal any important correlations between clinicopathological findings and galectin-3 and cyclin D1 expression and in non small cell lung cancer. We didn't observed also prognostic value of cyclin D1 or galectin-3 expression. But we showed higher cyclin D1 expression in galectin-3 negative tumor tissues. We revealed also differences in correlations between galectin-3 and cyclin D1 expression in two main histopathological types of NSCLC.

## Competing interests

The authors declare that they have no competing interests.

## Authors' contributions

MK collected informations about patients (clinicopathological findings, survival time), carried out immunohistochemical studies, performed statistical analysis and drafted manuscript. PP, AK and MG participated in collection of patient's data. RJ coordinated the study and improved manuscript. All authors read and approved the final manuscript.
